# Knockdown resistance (*kdr*) mutations within seventeen field populations of *Aedes albopictus* from Beijing China: first report of a novel V1016G mutation and evolutionary origins of *kdr* haplotypes

**DOI:** 10.1186/s13071-019-3423-x

**Published:** 2019-04-24

**Authors:** Xiaojie Zhou, Chan Yang, Nian Liu, Mei Li, Ying Tong, Xiaopeng Zeng, Xinghui Qiu

**Affiliations:** 1 Beijing Research Center for Preventive Medicine, Beijing Center for Disease Control and Prevention, Beijing, 100013 China; 2 State Key Laboratory of Integrated Management of Pest Insects and Rodents, Institute of Zoology, Chinese Academy of Sciences, Beijing, 100101 China; 3 University of Chinese Academy of Sciences, Beijing, 100049 China; 4 Institute of Physical Science and Information Technology, Anhui University, Anhui, 230039 China

**Keywords:** *Aedes albopictus*, Insecticide resistance, *kdr*, Voltage-gated sodium channel, Haplotype, Beijing

## Abstract

**Background:**

*Aedes albopictus* (Skuse) is an important vector of chikungunya, dengue, yellow fever and Zika viruses. In the absence of anti-viral medication and with limited availability of a commercial vaccine for public health use, vector control remains an effective means for reducing *Aedes*-borne disease morbidity. Knowledge about genetic mutations associated with insecticide resistance (IR) is a prerequisite for developing rapid resistance diagnosis, and the distribution and frequency of IR conferring mutations is important information for making smart vector control decisions.

**Methods:**

Partial DNA sequences of domain II and domain III of *Ae. albopictus* voltage gated sodium channel (*VGSC*) gene were amplified from a total of 426 individuals, collected from 17 sites in the Beijing municipality. These DNA fragments were sequenced to discover the possible genetic mutations mediating knockdown resistance (*kdr*) to pyrethroids. The frequency and distribution of *kdr* mutations were assessed in the 17 *Ae. albopictus* populations. The origin of *kdr* mutations was investigated by haplotype clarification and phylogenetic analysis.

**Results:**

Sequence alignments revealed the existence of multiple mutations (V1016G, I1532T, F1534S and F1534L) in VGSC. The highest frequency of the mutant 1016G allele (0.647) was found in Haidian, while 1016G was not detected in Huai Rou, Yan Qing, Ping Gu and Shun Yi. The frequency of 1532T was highest (0.537) in the population from the Olympic Forest Park (OFP, Chao Yang District), but not detectable in Huai Rou and Mi Yun. Two mutations were observed at codon 1534 with different distribution patterns: 1534L was only found in Tong Zhou (TZ) with a frequency of 0.017, while 1534S was distributed in TZ, OFP, Fang Shan, Da Xing and Shi Jing Shan with frequencies ranging from 0.019 (OFP) to 0.276 (TZ). One 1016G, one 1532T, one 1534L and two 1534S haplotypes were identified.

**Conclusions:**

Multiple mutations (V1016G, I1532T, F1534L/S) in VGSC were found in *Ae. albopictus* in Beijing. This represents the first report of V1016G in *Ae. albopictus*. Sequence alignment and phylogenetic analysis revealed multiple origins of 1534S. The spatial heterogeneity in distribution and frequency of *kdr* mutations calls for a site-specific strategy for the monitoring of insecticide resistance. The relatively high frequencies of V1016G warn of a risk of pyrethroid resistance in mosquitoes in the urban zones.

## Background

The Asian tiger mosquito *Aedes albopictus* is a major vector of four important arboviruses, chikungunya virus, dengue virus, yellow fever virus and Zika virus [1]. Although *Ae. albopictus* is native to Southeast Asia, the Western Pacific islands and islands of the Indian Ocean, it has gradually spread in recent decades [2]. The current worldwide distribution of *Ae. albopictus* greatly increases the risk of vector-borne disease outbreaks and poses a global threat to public health [3–5]. In China, *Ae. albopictus* is the primary vector of dengue fever [6–8] and is susceptible to Zika virus [9].

Vector control remains an important means for the prevention and control of vector-borne epidemics by reducing the density of vector insects [10, 11]. Control of *Aedes* vectors currently relies on application of insecticides and habitat management [12]. However, continuous and intensive use of insecticides in the fields or in domestic setting has artificially created a direct or indirect selection pressure on vector insects, leading to the development of insecticide resistance (IR) [1, 12].

There are two major mechanisms conferring insecticide resistance in *Aedes* mosquitoes. One is metabolic resistance, mainly mediated by P450 monooxygenases [13, 14], glutathione transferases (GST) [15] and esterases (Est) [16]. The other is called target resistance, which is caused by point mutations in target proteins of insecticides, such as voltage gated sodium channels (VGSC), acetylcholinesterases and gamma aminobutyric acid receptors [17].

Pyrethroids are the frequently used insecticides for the control of adult mosquitoes because of its low mammalian toxicity and high and rapid activity in insects. This class of insecticide has been widely used as indoor/outdoor residual or space sprays for mosquito control in China since the 1980s [18]. VGSC are the primary target of pyrethroids in insects. VGSC mutation mediated knockdown resistance (*kdr*) is the common and main cause of resistance to pyrethroids in insects. Previous studies on *Ae. aegypti* have documented several point mutations (V410L, S989P, I1011M/V, V1016G/I, I1532T, F1534S/L/C, D1763Y; *Musca domestica* numbering) of VGSC [19–28], most of which locate in the S6 segment of domain II (IIS6) and IIIS6. These mutations are usually confined to specific geographical areas, and vary in frequency and effect on resistance [12]. However, only a few including V410L, S989P, I1011M, V1016G and F1534C, alone or in combination, have been confirmed to be associated with resistant phenotypes or functionally corresponded well to reduce VGSC sensitivity to pyrethroids [19–26]. Co-occurrence of certain mutations is commonly associated with higher levels of resistance [28–30]. For example, the combination of S989P, V1016G and F1534C alters the sensitivity to permethrin and deltamethrin by 1100-fold and 90-fold, respectively [30].

By contrast, fewer molecular and functional studies have been conducted on *Ae. albopictus.* The first *kdr* mutation (F1534C) was discovered in a Singapore population [31], and two different mutations at codon 1534 (F1534S/L) were later described [32–35]. In addition, a mutation at codon 1532 (I1532T) was detected in Italy [33] and China [18, 34]. So far, although amino acid replacements at residues 1532 and 1534 of VGSC have been documented in Chinese *Ae. albopictus*, mutations at other IR-related loci have not been well examined.

Beijing is the capital of China with a large human population. To reduce the risk of vector-borne diseases, mosquito control programs have been implemented for 65 years (since the patriotic health campaign was initiated in 1952 [36]). In particular, pest (including mosquito, fly, rat and cockroach) control campaigns have been conducted in order to build “healthy district” in all 16 districts of Beijing Municipality in the last 10 years. It is very likely that insecticide-resistant alleles have been selected in the field due to the regular and continual application of pyrethroids. However, no comprehensive study on IR status and IR associated genetic mutation has been reported on field *Ae. albopictus* populations in Beijing. In this study, we sequenced partial DNA sequences of the *VGSC* gene from field-caught *Ae. albopictus* samples, and evaluated the frequencies of *kdr* alleles within seventeen *Ae. albopictus* populations. In addition, we attempted to examine the origin of *kdr* mutations by haplotype clarification and phylogenetic analysis.

## Methods

### Samples

*Aedes albopictus* samples were obtained from 17 sites located in 16 administrative districts of Beijing. Adult mosquitoes were captured by CDC light trap baited with CO_2_ from 16:00 to 19:00 h during July-September in 2016 or 2017. Basic information about the sample collection is described in Table 1. The *Aedes* samples provided by the Beijing Center for Disease Control and Prevention (CDC) were further identified by a PCR method described by Higa et al. [37] using species-specific primers based on the rDNA-ITS sequence. The molecularly re-confirmed *Ae. albopictus* specimens were then used for *kdr* genotyping.

**Table 1:** Brief information for *Ae. albopictus* collection in Beijing

Sampling site	District	Code	Environment	Coordinates	Date
Bei Shan	Chang Ping	CP	Forest park	40°14′9.11″N, 116°13′59.76″E	August 2017
Olympic Forest Park	**Chao Yang**	OFP	Forest park	40°0′54.22″N, 116°23′7.10″E	August 2017
Pan Jia Yuan Market	**Chao Yang**	PJY	Composite market	39°52′27.59″N, 116°27′6.85″E	August 2017
Rui Kang Jia Yuan	Da Xing	DX	Residential community	39°44′16.81″N, 116°20′9.13″E	July 2017
Bei Bing Ma Si Hu Tong	**Dong Cheng**	DC	Residential community	39°56′6.09″N, 116°23′53.41″E	July 2017
Yan Fang Lu	Fang Shan	FS	Residential community	39°43′18.36″N, 115°57′58.17″E	July 2017
Xin Fu Li Xiao Qv	**Feng Tai**	FT	Residential community	39°50′44.13″N, 116°23′14.27″E	August 2016
He Bei Cun, Shang Zhuang	**Hai Dian**	HD	Wetland park	40°01′59.71″N, 116°22′54.92″E	September 2016
Hong Luo Hui Yuan Gu,	Huai Rou	HR	Valley park	40°23′43.74″N, 116°34′39.38″E	August 2017
Lv Dao Jia Yan	Men Tou Gou	MTG	Lvdao community	39°56′32.70″N, 116°05′55.08″E	August 2017
Xi Weng Zhuang	Mi Yun	MY	Valley park	40°27′56.91″N, 116°53′23.26″E	August 2017
Ta Shan Jia Xiao	Ping Gu	PG	Driving school	40°05′49.90″N, 117°06′54.40″E	August 2017
Song Lin Gong Yuan	**Shi Jing Shan**	SJS	Forest park	39°54′15.66″N, 116°12′3.33″E	September 2017
Shi Yuan Dong She Qv	Shun Yi	SY	Residential community	40°06′33.87″N, 116°39′39.92″E	September 2017
Yun He Gong Yuan	Tong Zhou	TZ	Canal park	39°54′8.58″N, 116°40′20.92″E	July 2017
De Wai Xiao Qv	**Xi Cheng**	XC	Residential community	39°57′34.72″N, 116°22′48.49″E	July 2017
Shung Lu Xiao Qv	Yan Qing	YQ	Residential community	40°27′49.71″N, 115°59′48.57″E	August 2017

*Note*: The urban districts are in bold text

### Amplification and sequence analysis of *VGSC* fragments

Primers V2F (5′-GAC AAT GTG GAT CGC TTC CC-3′) and V2R (5′-GCA ATC TGG CTT GTT AAC TTG-3′) [31] were used to amplify the partial sequence of domain II covering codons encoding amino acid residues 989, 1011 and 1016. The reaction was done in a final volume of 30 μl, comprising 0.3 μl of Ex Taq DNA polymerase (Takara Bio, Dalian, China), 3 μl of 10× buffer, 2.4 μl of dNTP, 0.6 μl of 10 μM V2F, 0.6 μl of 10 μM V2R, 200 ng of DNA template, and ddH_2_O to make up to 30 μl. The reaction procedure was as follows: 94 °C for 5 min, followed by 38 cycles of 94 °C for 30 s, 60 °C for 30 s and 72 °C for 40 s, with a final extension at 72 °C for 10 min. The PCR products were gel-purified and directly sequenced from both directions using the forward (V2F) and reverse (V2R) primers, respectively.

Primers V3F (5′-GAG AAC TCG CCG ATG AAC TT-3′) and V3R (5′-TAG CTT TCA GCG GCT TCT TC-3′) [31] were used to amplify the fragment of domain III containing codons 1532 and 1534. The reaction system comprised 0.3 μl of Ex Taq DNA polymerase (Takara Bio), 3 μl of 10× buffer, 2.4 μl of dNTP, 1.2 μl of 10 μM V3F, 1.2 μl of 10 μM V3R, 200 ng of DNA template, and ddH_2_O, in a total volume of 30 μl. The reaction procedure was as follows: 94 °C for 5 min, followed by 38 cycles of 94 °C for 30 s, 58 °C for 30 s and 72 °C for 40 s, with a final extension at 72 °C for 10 min. The PCR products were sequenced using the reverse primer (V3R).

The sequences of the fragments of domain II (D2) and the fragments of domain III (D3) were aligned by MUSCLE v.3.8 [38], and nucleotide polymorphisms (SNPs) were documented.

### Haplotype identification

The haplotypes of *VGSC* alleles were identified by directly reading from homozygotes, or by splitting one from the other from heterozygotes carrying one-site variations, or by clone sequencing of heterozygotes with multiple-site variations. For clone sequencing, purified PCR products were ligated with the pEasy-T1 vector and transformed into competent cells of the *Escherichia coli* Trans 5α strain (Transgen, Beijing, China). Then, three to ten clones were sequenced. The sequences obtained from clone sequencing and from direct PCR product sequencing were cross-checked to clarify the two haplotypes in each heterozygote.

### Phylogenetic analysis

The intron + exon sequences of confirmed haplotypes were used for phylogenetic analysis by the maximum likelihood method based on the Tamura-Nei model [39] using MEGA v.7 [40].

## Results

### Genetic mutations in *Ae. albopictus VGSC*

**Fig. 1:**
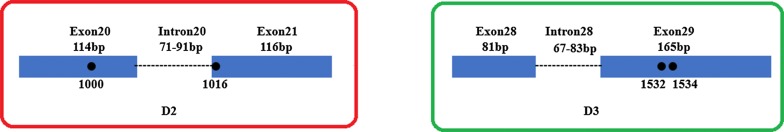
Schematic representation of the two regions of *Ae. albopictus VGSC* gene analyzed in this study. The intron-exon structure between the predicted initiation codon and stop codon is identified based on the genomic DNA sequence (MNAF02001058.1) and cDNA sequence (XM_019696540.1)

**Fig. 2:**
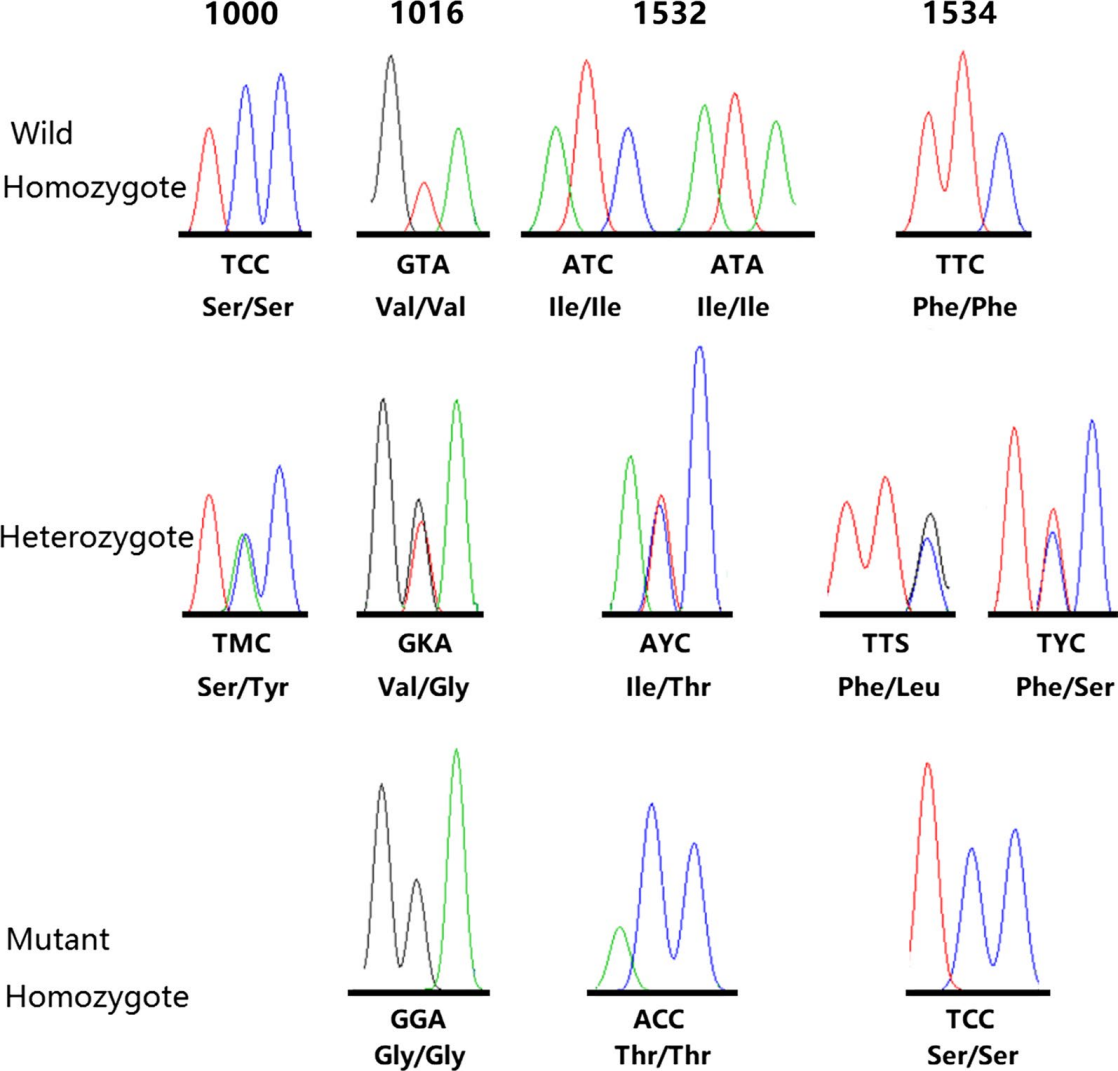
Example chromatograms showing the non-synonymous mutations identified in this study

The domain II fragment (D2) contained partial exon 20, the full intron 20 and partial exon 21 of the *Ae. albopictus VGSC* gene (Fig. 1). Two non-synonymous mutations, S1000Y (TCC to TAC) and V1016G (GTA to GGA), were detected (Fig. 2). 1000Y was found in seven individuals from TZ, and existed in the heterozygous form. 1016G was found in both heterozygotes and homozygotes.

The domain III fragment (D3) contained partial exon 28, the full intron 28 and partial exon 29 (Fig. 1). Three non-synonymous mutations I1532T (ATC or ATA to ACC), F1534S (TTC to TCC) and F1534L (TTC to TTG) were identified in exon 29 (Fig. 2). 1532T and 1534S existed in both heterozygous and homozygous forms. 1534L was only found in one heterozygote from TZ. The 1534C allele was not detected in our samples.

### Frequency and distribution of *kdr* mutations

**Fig. 3:**
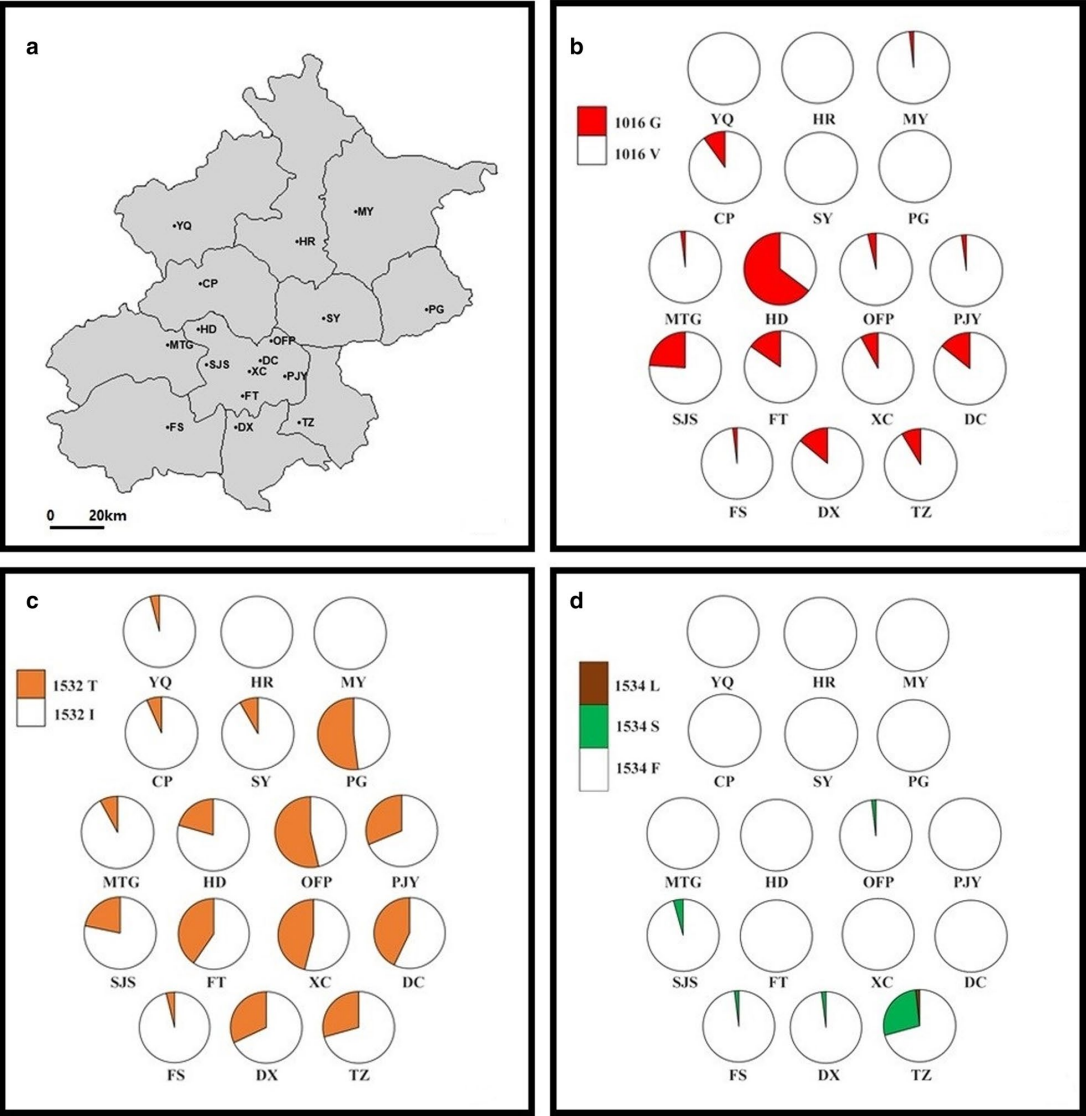
Distribution and frequency of *kdr* mutations in *Ae. albopictus* populations in Beijing. **a** Geographical position of the sampling sites. The shape file was downloaded from the GADM database of Global Administrative Areas (http://gadm.org/) on December 24, 2018. **b** 1016G mutation. **c** 1532T mutation. **d** 1534L/S mutations

In the examined 17 populations, IR-related (*kdr*) mutations at three loci (1016, 1532 and 1534) were detected (Table 2, Fig. 2). The difference in distribution of each *kdr* mutation is obvious in Fig. 3. For example, diverse mutations at multiple sites were detected in TZ, while *kdr* mutation was not observed in HR. 1016G was detected in 13 of the 17 populations with a frequency ranging from 0.019 (MY) to 0.647 (HD). Specifically, the frequency of the 1016G allele was relatively high in the urban zones (e.g. DC, HD, SJS, FT), but low in rural areas. In particular, 1016G was not detected in four rural districts HR, YQ, PG and SY.

**Table 2:** Frequency and distribution of *VGSC* alleles in Beijing *Ae. albopictus* grouped by the genotypes of codons 1016, 1532 and 1534

Location	*n*	1016			1532			1534			
		V/V	V/G	G/G	I/I	I/T	T/T	F/F	F/S	F/L	S/S
CP	30	26	2	2	26	4		30			
CY(PJY)	24	23	1		11	11	2	24			
CY(OFP)	27	25	2		7	11	9	26	1		
DC	28	20	8		8	16	4	28			
DX	25	19	5	1	12	10	3	24	1		
FS	26	25	1		25		1	25	1		
FT	26	19	6	1	11	9	6	26			
HD	17		12	5	10	7		17			
HR	27	27			27			27			
MTG	25	24	1		21	4		25			
MY	27	26	1		27			27			
PG	25	25			8	8	9	25			
SJS	23	14	7	2	14	8	1	21	2		
SY	18	18			15	3		18			
TZ	29	24	5		13	15	1	13	14	1	1
XC	25	21	4		8	11	6	25			
YQ	24	24			22	2		24			
Total	426	360	55	11	265	119	42	405	19	1	1

*Abbreviation*: *n*, the size of each population

1532T was found in 15 populations, with the highest frequency of 0.537 being in OFP. Two substitutions at codon 1534 (i.e. 1534L and 1534S) were detected in TZ, with the frequency of 1534S (0.276) being higher than that of 1534L (0.017). 1534S was also detected in OFP, FS, DX and SJS at low frequencies (< 0.050).

### Frequency and distribution of triple-locus genotype combinations in the *Ae. albopictus VGSC* gene

From our samples in a total of 426 individuals, we found 11 types of triple-locus genotype combinations in *Ae. albopictus* (Table 3). The number of triple-locus genotype combinations observed in each sampling site ranged from 1 (HR) to 10 (TZ). Notably, similar genotype distribution patterns were observed between DC and XC, and between DX and SJS. In addition, two combinations (Type 4 and Type 8) were uniquely present in TZ.

**Table 3:** Frequency distributions of triple-locus genotype combinations in 17 *Ae. albopictus* populations collected from Beijing, China

Type	Loci			Population																
	1016	1532	1534	CP	CY (OFP)	CY (PJY)	DC	DX	FS	FT	HD	HR	MTG	MY	PG	SJS	SY	TZ	XC	YQ
Type 1	V/V	I/I	F/F	0.733	0.185	0.417	0.179	0.320	0.885	0.308		1.000	0.800	0.963	0.320	0.261	0.833	0.103	0.240	0.917
Type 2	**G**/**G**	I/I	F/F	0.067				0.040		0.038	0.294					0.087				
Type 3	V/V	**T**/**T**	F/F		0.333	0.083	0.143	0.120	0.038	0.231					0.360	0.043		0.034	0.240	
Type 4	V/V	I/I	**S**/**S**															0.034		
Type 5	V/**G**	I/I	F/F	0.067	0.037	0.042	0.107	0.120	0.038	0.077	0.294		0.040	0.037		0.261		0.103	0.080	
Type 6	V/V	I/**T**	F/F	0.133	0.407	0.458	0.393	0.280		0.192			0.160		0.320	0.217	0.167	0.172	0.360	0.083
Type 7	V/V	I/I	F/**S**						0.038									0.138		
Type 8	V/V	I/I	F/**L**															0.034		
Type 9	V/**G**	I/**T**	F/F				0.179	0.080		0.154	0.412					0.043		0.034	0.080	
Type 10	V/V	I/**T**	F/**S**					0.040								0.087		0.310		
Type 11	V/**G**	I/I	F/**S**		0.037													0.034		

*Note*: The mutant amino acid is in bold

Triple-locus wild homozygotes (Type 1) were observed in 16 populations. This type occurred at high frequencies in HR (1.000), MY (0.963), YQ (0.917), FS (0.885), SY (0.833), MTG (0.800) and CP (0.733), but at low frequencies in TZ and OFP. The presence of single mutation was observed at loci 1016, 1532 and 1534 respectively (Types 2, 3, 4, 5, 6, 7 and 8). The combinations (Type 5 and Type 6) heterozygous at one IR-related locus (either 1016 or 1532) were widely distributed. Combinations (Types 9, 10 and 11) heterozygous at two of the three *kdr* loci (i.e. 1016 + 1532, 1016 + 1534, or 1532 + 1534) were detected. Single mutant homozygotes 1016GG (Type 2), 1532TT (Type 3) and 1534SS (Type 4) were present, while there were no double-loci mutant homozygotes or triple-locus mutant individuals.

### Nucleotide polymorphism and haplotype diversity of the *Ae. albopictus VGSC* gene

*VGSC* haplotypes were experimentally clarified from our samples. Seventeen haplotypes were identified based on 200 sequences of D2 from 151 individuals in this study (Table 4). Sequence analysis showed that there were length polymorphisms in intron-20 among these haplotypes; the shortest intron was 71 bp, while the longest one was 91 bp. In the exon region of D2 (230 bp in length), 11 sites were polymorphic. The nucleotide variations resulted in two amino acid substitutions (S1000Y and V1016G) (Fig. 2).

**Table 4:** Haplotype identification based on D2 sequence in *Aedes albopictus*

Haplotype	GenBank ID	Intron-20 length (bp)	Residue 1000	Residue 1016	Best-hit ranked by E-value (cover, identity)
D2H01	MK201602	82	Ser (S)	Val (V)	KC152045 (100%, 93%)
D2H02	MK201603	91	Ser (S)	Val (V)	KC152045 (100%, 100%)
D2H03	MK201604	88	Ser (S)	Val (V)	KC152045 (100%, 96%)
D2H04	MK201605	91	Ser (S)	Val (V)	KC152045 (100%, 99%)
D2H05	MK201606	89	Ser (S)	Val (V)	KC152045 (100%, 96%)
D2H06	MK201607	90	Ser (S)	Val (V)	KC152045 (100%, 95%)
D2H07	MK201608	89	Ser (S)	Gly (G)	KC152045 (100%, 95%)
D2H08	MK201609	90	Ser (S)	Val (V)	KC152045 (100%, 93%)
D2H09	MK201610	91	Ser (S)	Val (V)	KC152045 (100%, 99%)
D2H10	MK201611	71	Tyr (Y)	Val (V)	KC152045 (100%, 90%)
D2H11	MK201612	90	Ser (S)	Val (V)	KC152045 (100%, 95%)
D2H12	MK201613	91	Ser (S)	Val (V)	KC152045 (100%, 97%)
D2H13	MK201614	91	Ser (S)	Val (V)	KC152045 (100%, 99%)
D2H14	MK201615	90	Ser (S)	Val (V)	KC152045 (100%, 94%)
D2H15	MK201616	91	Ser (S)	Val (V)	KC152045 (100%, 99%)
D2H16	MK201617	90	Ser (S)	Val (V)	KC152045 (100%, 93%)
D2H17	MK201618	82	Ser (S)	Val (V)	KC152045 (100%, 94%)

Similarly, 19 haplotypes were identified based on 165 sequences of D3 from 124 individuals (Table 5). Length polymorphisms were also observed in intron-28 among these haplotypes. The shortest intron was 67 bp, while the longest one was 83 bp. In the coding region (246 bp in length), there were 10 polymorphic sites, leading to non-synonymous mutations at codons 1532 and 1534 (Fig. 2).

**Table 5:** Haplotype identification based on D3 sequence in *Aedes albopictus*

Haplotype	GenBank ID	Intron-28 length (bp)	Residue 1532	Residue 1534	Best-hit ranked by E-value (cover, identity)
D3H01	MK201619	68	Thr (T)	Phe (F)	MH384957 (92%, 100%)
D3H02	MK201620	83	Ile (I)	Phe (F)	KC152046 (100%, 100%)
D3H03	MK201621	83	Ile (I)	Ser (S)	MH384960 (92%, 100%)
D3H04	MK201622	83	Ile (I)	Phe (F)	MH384960 (92%, 99%)
D3H05	MK201623	83	Ile (I)	Phe (F)	KC152046 (100%, 99%)
D3H06	MK201624	68	Ile (I)	Phe (F)	MH384953 (92%, 99%)
D3H07	MK201625	83	Ile (I)	Phe (F)	KC152046 (100%, 99%)
D3H08	MK201626	68	Ile (I)	Phe (F)	MH384957 (92%, 99%)
D3H09	MK201627	83	Ile (I)	Phe (F)	KC152046 (100%, 98%)
D3H10	MK201628	83	Ile (I)	Phe (F)	KC152046 (100%, 98%)
D3H11	MK201629	70	Ile (I)	Phe (F)	KC152046 (100%, 94%)
D3H12	MK201630	83	Ile (I)	Leu (L)	KC152046 (100%, 99%)
D3H13	MK201631	68	Ile (I)	Ser (S)	MH384956 (92%, 100%)
D3H14	MK201632	72	Ile (I)	Phe (F)	KC152046 (100%, 94%)
D3H15	MK201633	68	Ile (I)	Phe (F)	MH384953 (92%, 99%)
D3H16	MK201634	68	Ile (I)	Phe (F)	MH384953 (92%, 99%)
D3H17	MK201635	68	Ile (I)	Phe (F)	MH384953 (92%, 99%)
D3H18	MK201636	67	Ile (I)	Phe (F)	MH384957 (92%, 97%)
D3H19	MK201637	83	Ile (I)	Phe (F)	KC152046 (100%, 97%)

### Haplotypes related to insecticide resistance

By investigating 32 out of the 66 individuals harboring the 1016G substitution, only one identical 1016G haplotype (D2H07) was identified. Similarly, the 1532T mutation was detected in 161 mosquitoes, and only one identical 1532T haplotype (D3H01) was found after analyzing 35 homozygotes and 30 heterozygotes sampled across populations.

**Fig. 4:**
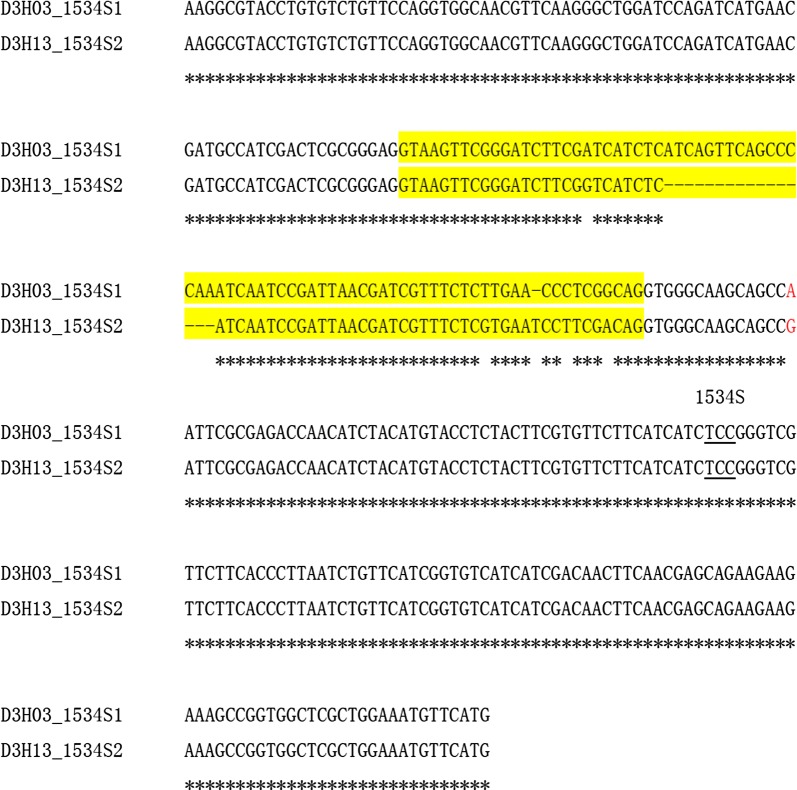
Alignment of nucleotide sequences of two 1534S haplotypes. The intron is highlighted in yellow. The codon encoding the 1534 residue is underlined. Asterisks indicate identical nucleotides

The 1534S mutation was found in 20 individuals, of which 16 (12 from TZ, one each from DX, FS, OFP and SJS) were used for haplotype identification. Two different 1534S haplotypes (D3H03 and D3H13) were found. Sequence alignment showed that the two haplotypes differed in both exon and intron regions (Fig. 4). Interestingly, their distribution was different: D3H03 was found only in TZ, while D3H13 was exclusively detected in the other four sites (DX, FS, OFP and SJS). A BLAST search showed that the sequence of D3H03 was identical to that of MH384960.1 (*Ae. albopictus* voucher HK48 voltage-gated sodium channel gene), and D3H13 was identical to MH384956.1 (*Ae. albopictus* voucher YP01 voltage-gated sodium channel gene) [18] and MH384954.1 (*Ae. albopictus* voucher HZ45 voltage-gated sodium channel gene) [18].

**Fig. 5:**
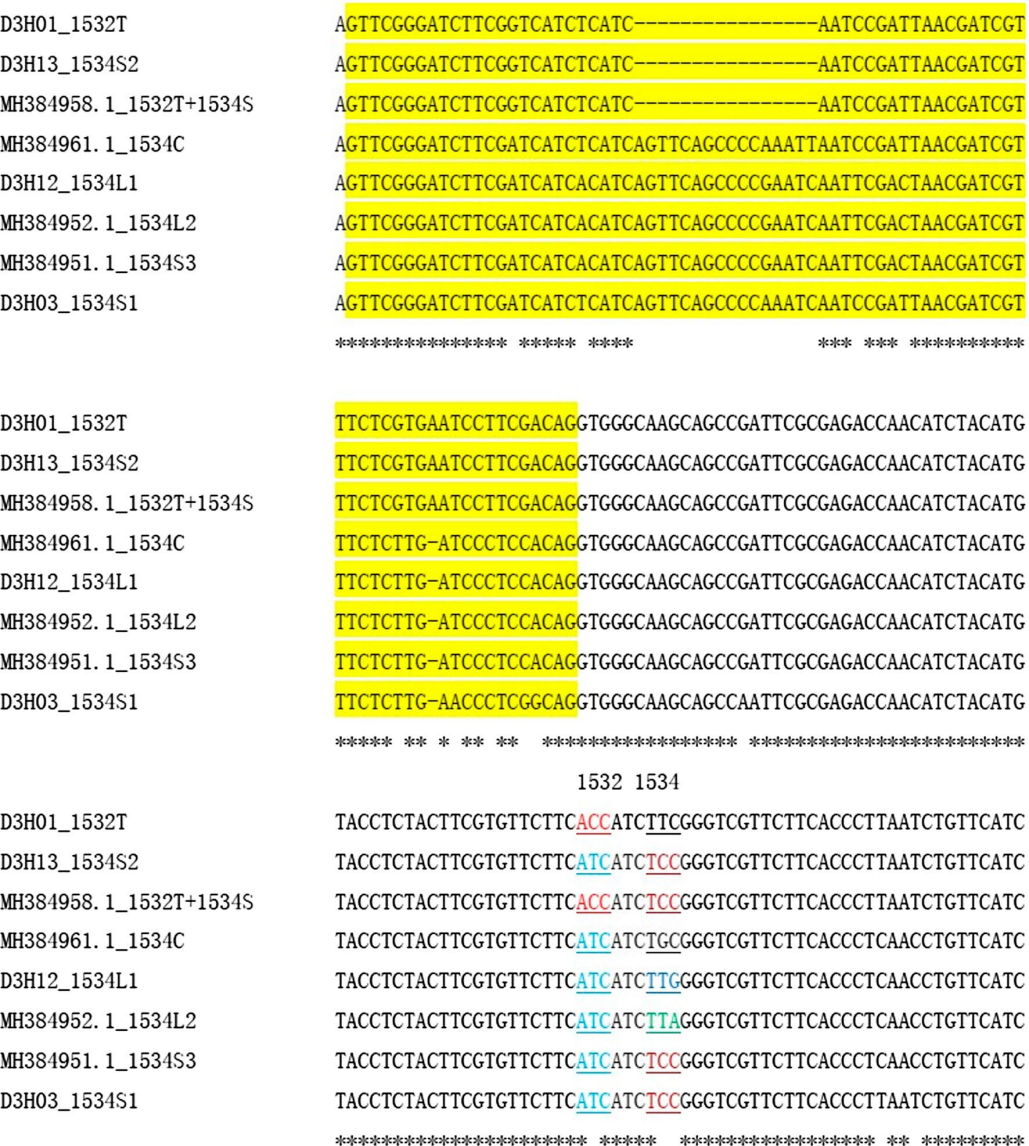
Alignment of nucleotide sequences of mutant *VGSC* haplotypes. The 5' and 3' regions that are identical are not presented in the figure. The intron is highlighted in yellow. The codons encoding 1532 and 1534 residues are underlined. Asterisks indicate identical nucleotides

There was only one individual that harbored the 1534L in our samples, leading to the identification of one 1534L haplotype (D3H12). D3H12 was highly similar to MH384952.1 (*Ae. albopictus* voucher JN08 voltage-gated sodium channel gene) [18]; they encoded the same amino acid sequence with only one variation (G/A) in the third position of codon 1534 (Fig. 5). No haplotype harboring both 1532 and 1534 mutations was observed after examining all 12 specimens that carried both 1532T and 1534S.

### Evolutionary analysis of *kdr* haplotypes

**Fig. 6:**
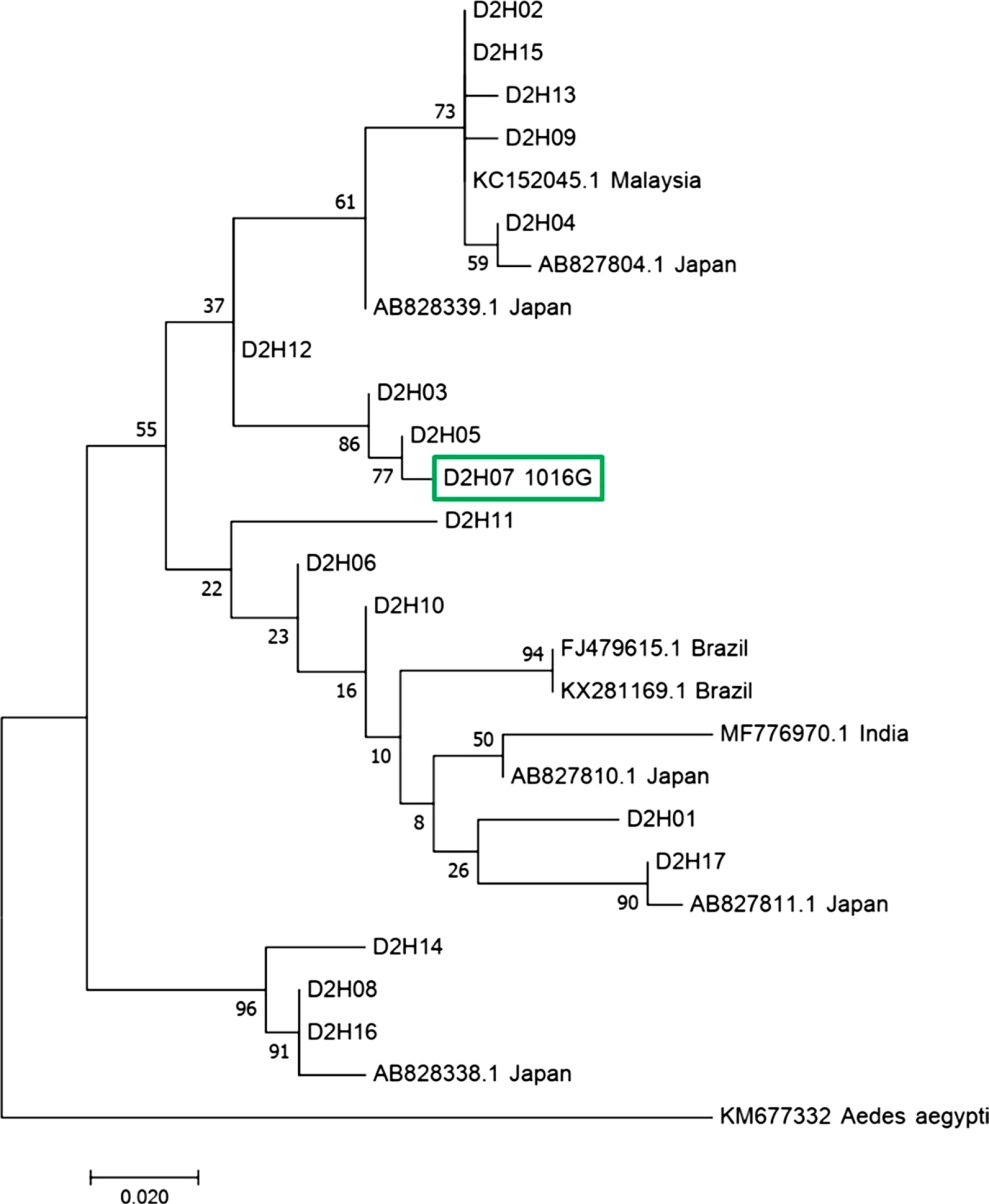
Molecular phylogenetic analysis by the maximum likelihood method based on D2 sequences. The GenBank accessions for the sequences identified in this study (named as D2Hx) are provided in Table 4. The tree with the highest log likelihood (−917.40) is shown. The percentage of trees in which the associated taxa clustered together is shown next to the branches. Initial tree(s) for the heuristic search were obtained automatically by applying Neighbor-Join and BioNJ algorithms to a matrix of pairwise distances estimated using the Maximum Composite Likelihood (MCL) approach, and then selecting the topology with the superior log-likelihood value. The tree is drawn to scale, with branch lengths measured in the number of substitutions per site. The analysis involved 27 nucleotide sequences. There were a total of 317 positions in the final dataset

**Fig. 7:**
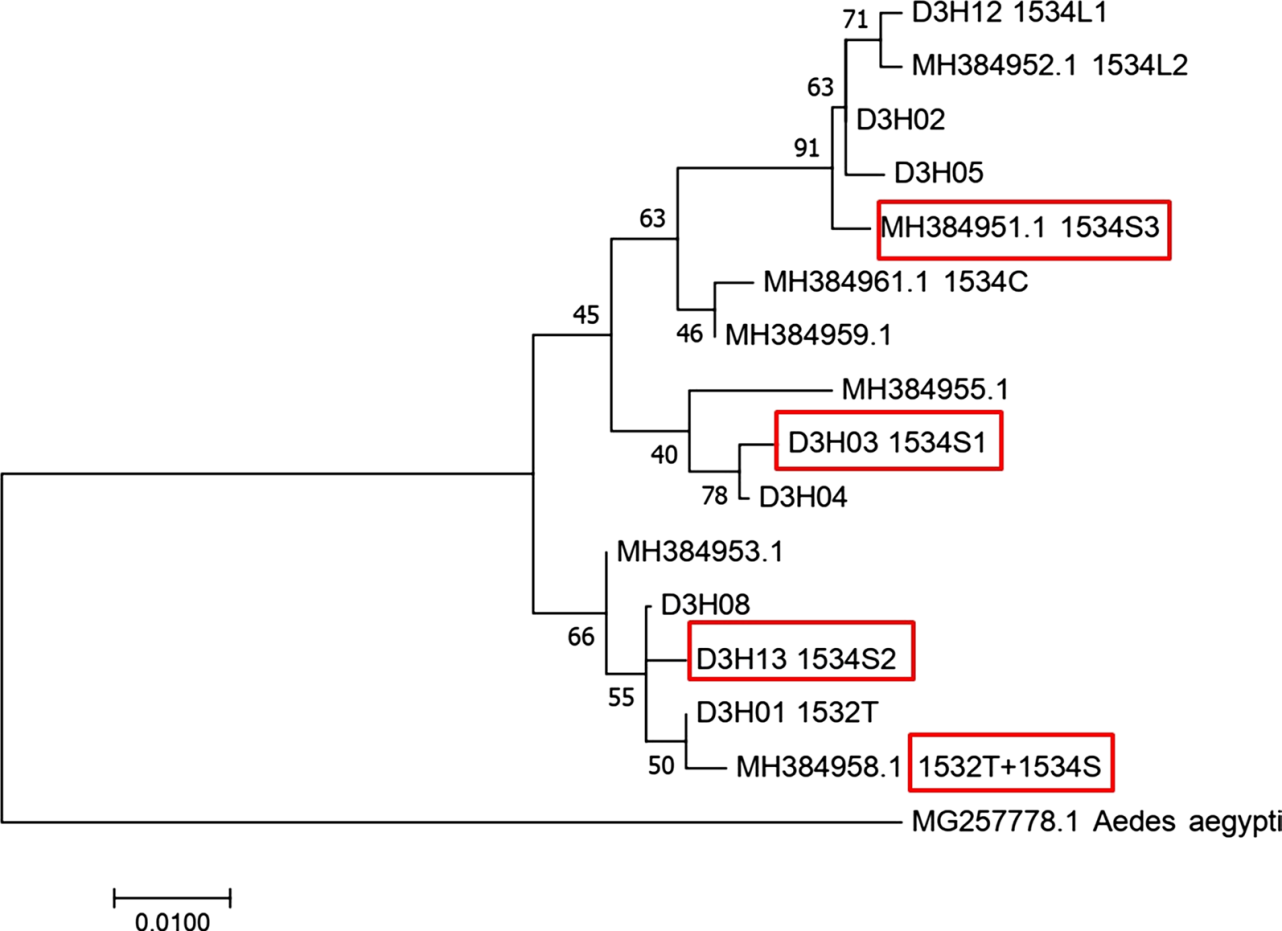
Molecular phylogenetic analysis by the Maximum Likelihood method based on D3 sequences. The GenBank accessions for the sequences identified in this study (named as D3Hx) are provided in Table 5. The evolutionary history was inferred by using the maximum likelihood method based on the Tamura-Nei model [1]. The tree with the highest log-likelihood (−744.91) is shown. The percentage of trees in which the associated taxa clustered together is shown next to the branches. Initial tree(s) for the heuristic search were obtained automatically by applying Neighbor-Join and BioNJ algorithms to a matrix of pairwise distances estimated using the Maximum Composite Likelihood (MCL) approach, and then selecting the topology with the superior log-likelihood value. The tree is drawn to scale, with branch lengths measured in the number of substitutions per site. The analysis involved 16 nucleotide sequences. There were a total of 306 positions in the final dataset

To investigate the phylogenetic relationship of *kdr* haplotypes with other wild counterparts, two maximum likelihood trees were constructed based on terminal-truncated D2 and D3 sequences, and some corresponding sequences retrieved from GenBank (Figs. 6, 7). Figure 6 shows that the D2H07 harboring 1016G substitution is closely clustered with D2H05 and D2H03 in an independent branch. Inferred from the tree in Fig. 7, haplotypes carrying either 1532T or 1534L were clustered in one of the two major clades, respectively, while those with a 1534S mutation were divided into two different major clades. Although belonging to the same major clade, the two 1534L haplotypes were closely clustered, while 1534S2 and 1534S3 were located in different sub-clades.

**Fig. 8:**
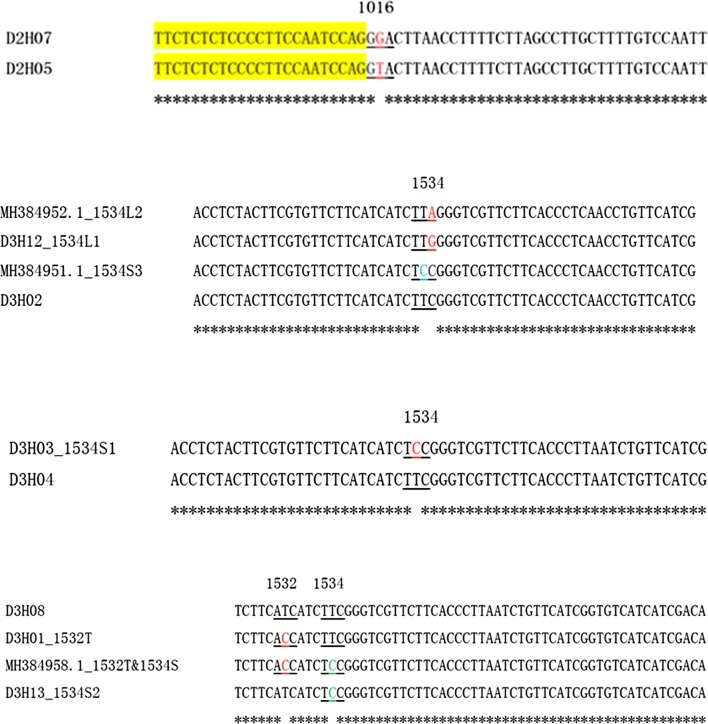
Alignment of nucleotide sequences of *kdr* haplotypes. The sequences (*c.*300 bp in length) in each alignment are highly similar, differing by 1 or 2 nucleotides. Only the region with variations is showed in the figure. The intron is highlighted in yellow. The codons encoding the insecticide resistance related amino acid residues are underlined. Asterisks indicate identical nucleotides

To track the evolutionary origin of the *kdr* haplotypes, sequence alignments of the closely clustered haplotypes were conducted. The results, shown in Fig. 8, indicate that the resistant haplotypes D2H07 (1016G) might be derived from the wild one (D2H05) through one mutational step. Similarly, one nucleotide alternation in D3H02 could create three mutants (1534L1, 1534L2 and 1534S3), while one nucleotide replacement in D3H04 could lead to the formation of 1534S1 (Fig. 8). The double-mutation (1532T + 1534S) haplotype of *Ae. albopictus* voucher YP37 voltage-gated sodium channel gene (MH384958.1) [18] differed from either D3H01 (1532T) or D3H13 (1534S2) by only one nucleotide (Fig. 8), suggesting that these *kdr* haplotypes share a common wild ancestor (D3H08).

## Discussion

This study represents a comprehensive investigation of *kdr-*related mutations in *Ae. albopictus* mosquitoes across Beijing. Besides the known mutations (I1532T, F1534S/L), we identified a novel V1016G substitution in *Ae. albopictus.* The information about the frequency and distribution of *kdr* mutations could help us to understand the genetic mechanism of insecticide resistance in *Ae. albopictus.* The defined *VGSC* haplotypes are useful for tracing the evolutionary origin of *kdr* mutations.

Sequence alignments of D2 revealed a mutation at loci 1016 (V1016G) in our *Ae. albopictus* samples. Although a conserved V1016G *kdr* mutation has been documented in *Aedes aegypti* [41], to our knowledge, this is the first identification of the V1016G mutation in *Ae. albopictus.* Notably, 1016G was detected in 13 of the 17 populations with the highest frequency of 0.65 (65%), and 1016G/G homozygotes were found in five populations. These results indicate that 1016G resistant allele frequently occurs at least in Beijing. A literature review showed that most previous studies focused on genetic mutations in domain III [18, 34], the occurrence of 1016G in *Ae. albopictus* is probably underestimated. Given that V1016G substitution in *Aedes aegypti* can lead to insensitivity to both Type I and Type II pyrethroids, a global survey of the distribution of the V1016G mutation in *Ae. albopictus* is strongly suggested.

I1532T mutation has been previously reported in *Ae. albopictus* from Italy [33] and different areas of China [18, 34, 42]. In this study, I1532T mutation was observed in 161 individuals from 15 of 17 populations with a frequency ranging from 0.038 to 0.537. These findings demonstrate that 1532T has a widespread geographical distribution in Chinese *Ae. albopictus*. Thus far, the association of phenotype with I1532T substitution remains unknown. Given the prevalence of this allele in China, it is important to pursue further studies on the biological and pharmacological effects of I1532T.

Different from 1016G and 1532T, mutations at codon 1534 are multiple. Two of the three previously documented amino acid substitutions at the 1534 locus (1534L and 1534S) were detected in Beijing. Notably, only one of 426 individuals was found to carry 1534L, suggesting that this mutation has just emerged in or was recently introduced into Beijing. Interestingly, the nucleotide alterations leading to F1534L varied among populations: a transversion at the third position occurred in our specimen (TTC to TTG), and another different transversion at the same position (i.e. TTC to TTA) was observed in mosquitoes from the JN population in a study covering five field populations from China [18], while the F1534L mutation identified in a survey in the USA resulted from a nucleotide transition at the first position (TTC to CTC) [32]. The 1534C detected in Singapore [31], Greece [33], Brazil [43], India [44] and even south China [18, 42], was not found in our samples. In contrast, 1534S mutation is widely distributed in China: it has been found in samples from southern, eastern, central and northern China in addition to this study [18, 42].

This study reveals obvious spatial heterogeneity of *kdr* mutations within Beijing *Ae. albopictus* populations. The number of mutations and frequency shows considerable geographical variations (Tables 2, 3; Fig. 3). Such an irregular pattern reflects that factors affecting the evolution and distribution of *kdr* mutations in *Ae. albopictus* are complex. Overall, 1016G and 1532T alleles were commonly present in Beijing, with 1534S occurring mainly in TZ (Fig. 4). Compared to the rural districts, higher frequencies of 1016G were detected in the urban districts (DC, XC, CY, HD, FT, SJS), which is in-line with more intensive insecticide use and higher residential density.

Given that a significant association of V1016G and F1534S/C mutations in *Aedes* VGSC with resistance phenotypes has been established by a large amount of susceptibility bioassay or/and electrophysiological evidence [18–22, 24–26, 29, 33–35], the distribution and frequency of conserved *kdr* mutations (Fig. 3) could serve to predict the status of insecticide resistance in Beijing *Ae. albopictus.* The relatively high frequency of 1016G in the urban districts (especially in HD), and the prevalent co-occurrence of 1016G and 1534S in TZ (Fig. 4), strongly indicates a risk of pyrethroid resistance in these areas. In addition, it is worth noting that 1016G homozygotes were present in CP, DX, FT and SJS, strongly suggesting a pyrethroid resistance risk. Unfortunately, since no parallel susceptibility bioassays were performed on the samples used for genotyping, we could not establish a clear association of IR levels with specific *kdr* alleles. However, a loss of susceptibility to beta cypermethrin was observed in HD, OFP and TZ (personal communications).

The results from phylogenetic analysis and alignments of DNA sequences of wild and mutant haplotypes may help trace the origin of *kdr* mutations. Considering that only one 1016G and one 1532T haplotype was discovered, we are unable to determine if 1016G or 1532T is singly or multiply originated. To determine the number of origins of 1016G or 1532T alleles, examination of a sufficient number of samples from a broad geographical range is required. However, the sequence alignments (Fig. 8) suggest that haplotypes D2H07 (1016G) and D3H01 (1532T) may have evolved from wild haplotypes D2H05 and D3H08, respectively.

Regarding codon 1534, multiple mutations have been documented. Moreover, it is already known that nucleotide mutations may happen at all the three positions of codon 1534 (i.e. T to C at the first position, T to G or C at the second position, and C to G or A at the third position), which leads to three different amino acid substitutions (F1534C/L/S). These observations indicate that codon 1534 is sensitive to mutation. The cross-continent distribution of conserved F1534C/L/S mutation may reflect the importance of this amino acid residue in adaptating to pyrethroid selection pressure. The large difference in DNA sequence between 1534S1 and 1534S2 (Fig. 4) makes us conclude that 1534S has multiple independent origins. By contrast, the extremely high similarity among 1534L1, 1534L2 and 1534S3 (Fig. 8) strongly suggests that these *kdr* haplotypes share a common origin. Similarly, we hypothesize that the three *kdr* haplotypes 1534S2 and 1532T (D3H01), and 1532T and 1534S (MH384958) may evolve from a common susceptible progenitor.

This work focused on *kdr* mutation. Other mechanisms such as enhanced detoxification of pyrethroids may possibly contribute to IR in field populations; therefore, further work is required to gain a full understanding about the status of pyrethroid resistance and underlying mechanisms in Beijing *Ae. albopictus* populations. Such studies are in our project list.

## Conclusions

This study delineates the distribution of *kdr* mutations in the dengue vector *Ae. albopictu*s across Beijing, China. Amino acid substitutions at multiple sites (1000, 1016, 1532, 1534) and two variations at codon 1534 have been documented. Phylogenetic analysis and sequence alignments have demonstrated multiple origins of 1534S. The presence of 1016G and 1534S/L alleles indicates a strong risk of pyrethroid resistance in Beijing *Ae. albopictu*s. These findings highlight the importance to monitor and quantify the level of pyrethroid resistance in field mosquitoes in the urban districts. Taking action to limit the spread of *kdr* alleles into the rural areas would be helpful to prevent the development of insecticide resistance.

## Abbreviations

IR: insecticide resistance
*kdr*: knockdown resistance
PCR: polymerase chain reaction
VGSC: voltage-gated sodium channel(s)
